# RGB image-based method for phenotyping rust disease progress in pea leaves using R

**DOI:** 10.1186/s13007-023-01069-z

**Published:** 2023-08-21

**Authors:** Salvador Osuna-Caballero, Tiago Olivoto, Manuel A. Jiménez-Vaquero, Diego Rubiales, Nicolas Rispail

**Affiliations:** 1 Institute for Sustainable Agriculture, CSIC, Av. Menéndez Pidal s/n 14004, Córdoba, Spain; 2 Department of Plant Science, Federal University of Santa Catarina, Florianópolis, 88034-000 SC Brazil

**Keywords:** Disease resistance, Image analysis, Pea, Phenotyping, Phytopathometry, Rust

## Abstract

**Background:**

Rust is a damaging disease affecting vital crops, including pea, and identifying highly resistant genotypes remains a challenge. Accurate measurement of infection levels in large germplasm collections is crucial for finding new resistance sources. Current evaluation methods rely on visual estimation of disease severity and infection type under field or controlled conditions. While they identify some resistance sources, they are error-prone and time-consuming. An image analysis system proves useful, providing an easy-to-use and affordable way to quickly count and measure rust-induced pustules on pea samples. This study aimed to develop an automated image analysis pipeline for accurately calculating rust disease progression parameters under controlled conditions, ensuring reliable data collection.

**Results:**

A highly efficient and automatic image-based method for assessing rust disease in pea leaves was developed using R. The method’s optimization and validation involved testing different segmentation indices and image resolutions on 600 pea leaflets with rust symptoms. The approach allows automatic estimation of parameters like pustule number, pustule size, leaf area, and percentage of pustule coverage. It reconstructs time series data for each leaf and integrates daily estimates into disease progression parameters, including latency period and area under the disease progression curve. Significant variation in disease responses was observed between genotypes using both visual ratings and image-based analysis. Among assessed segmentation indices, the Normalized Green Red Difference Index (NGRDI) proved fastest, analysing 600 leaflets at 60% resolution in 62 s with parallel processing. Lin’s concordance correlation coefficient between image-based and visual pustule counting showed over 0.98 accuracy at full resolution. While lower resolution slightly reduced accuracy, differences were statistically insignificant for most disease progression parameters, significantly reducing processing time and storage space. NGRDI was optimal at all time points, providing highly accurate estimations with minimal accumulated error.

**Conclusions:**

A new image-based method for monitoring pea rust disease in detached leaves, using RGB spectral indices segmentation and pixel value thresholding, improves resolution and precision. It rapidly analyses hundreds of images with accuracy comparable to visual methods and higher than other image-based approaches. This method evaluates rust progression in pea, eliminating rater-induced errors from traditional methods. Implementing this approach to evaluate large germplasm collections will improve our understanding of plant-pathogen interactions and aid future breeding for novel pea cultivars with increased rust resistance.

**Supplementary Information:**

The online version contains supplementary material available at 10.1186/s13007-023-01069-z.

## Background

Rusts are a group of plant diseases caused by species of the *Pucciniales* order which is one of the largest orders of plant fungal pathogens comprising more than 8,000 species [1]. They are obligate biotrophs that compromise yields of important crops worldwide and exhibit complex lifecycles with up to five different stages (i.e., pycnidial, aecial, uredial, telial, and basidial stages) [2]. Rust lifecycle begins when the spores, carried by wind or water, germinate and infect the aerial tissue of the host. Once inside the plant, it produces specialized structures called haustoria mother cells, which penetrate the plant cells via a neckband, and form haustoria to extract nutrients. Then, the fungus produces secondary spores, which can spread to other parts of the same plant or to new host plants. This infection cycle and spore production can be repeated several times along the cropping season, leading to the development of visible symptoms such as yellowing, spotting or rust-coloured pustules on the leaves, stems, or fruit of the host, depending on the rust species or host reaction [2, 3]. In many cases two taxonomically unrelated hosts are required to complete the life cycle. Different species of rust fungi have different host ranges, but many can infect a wide variety of plant species within a particular plant family or group, hindering their management in the field [4]. In pea (*Pisum sativum* L.), a valuable, versatile, and inexpensive protein source for human food and animal feed [5], rust is a major disease spread worldwide [6]. Two rust species, *Uromyces pisi* (Pers.) (Wint.) and *U. viciae-fabae* (Pers. de Bary) [7], have been described as causal agent of pea rust. The uredial stage of *U. pisi* produces the infective structures that affects pea crops in temperate regions while in warmest countries the aecial stage of *U. viciae-fabae* is the epidemic one [8]. Although agronomical practices and chemical control of pea rusts have been explored to reduce their incidence [9–15], the use of resistant cultivars is considered as the most effective, economic, and eco-friendly strategy for rust control [16]. To face the challenge of developing new rust resistant varieties, the reference genomes recently available provide important resources for pea breeding [17–20]. The constant reduction in sequencing cost coupled with the technological advances that refine marker-trait association and genome editing approaches are expected to boost future development of pea resistance breeding. However, these methods need to be fed with detailed and accurate phenotypic data to guide breeding and deepen our understanding of the genetic variations controlling complex traits, such as rust disease resistance. Phenotyping is therefore becoming the main bottleneck for breeding. It is particularly challenging when assessment of very large collection of several thousand lines which is the typical size of nested association mapping (NAM) populations [21–23]. It is therefore urgent to improve and optimize the available methods of phenotyping.

The phenotypic characterization of pea response to rust has relied on disease assays conducted under controlled or field condition, in seedlings or adult plants, and with natural or artificial infestation. In these assays, disease was evaluated by measuring qualitative and/or quantitative measurements. Qualitative assessment of rust disease, known as infection type (IT) usually use a scale ranging from 0 to 4, as described by Stakman et al. in wheat [24]. The IT depends on the host reaction to the pathogen. This reaction could be incompatible, when the host shows no symptoms or develops a hypersensitive response, or compatible when typical rust pustule develops on the susceptible host [4]. Quantitative assessment of rust symptoms is conventionally assessed as a visual estimation of the percentage of leaf area covered by rust pustules (disease severity, DS). This can be decomposed in more detailed components such as the infection frequency (IF) and colony size (CS). IF is the number of lesions (herein, pustules) within a limited area, usually 1 cm^2^. These parameters defined as objective are weakly affected by user bias but highly time consuming when screening large germplasm collections. Contrary to IF or CS, DS is a subjective parameter highly dependent on the user interpretation that requires specialized training [25]. DS is also affected by IT, so the user can confound the area surrounding pustules that sometimes develop chlorotic/necrotic regions. In several foliar diseases, standard area diagrams (SAD) can offer increased precision over DS calculations [26–28]. However, SAD are not readily available for pea rust.

Traditionally, qualitative, and quantitative measurements have been performed to better understand the resistance mechanisms that operate in pea-rust pathosystem, together with other ones considering the pustule size [29–31]. Periodical evaluation of these quantitative parameters allows to estimate disease progression factors such as the Area Under Disease Progress Curve (AUDPC), the Latency Period (LP_50_) and the Monocyclic Disease Progress rate (MDPr) [32, 33]. Through these factors, it is possible to capture most of the complexity of rust disease evolution.

Little advances have been achieved toward automatization of pea rust phenotyping in comparison with other aerial fungal pathosystems, for which many platforms and methodologies have been developed to increase accuracy and precision of disease estimation including from other fungal pathogens in legumes [34] to bacterial pathogens in citrus plants [35, 36]. Among these so-called high-throughput methods, development of image-based phenotyping techniques has largely increased in the last decade partly thanks to the decrease in imaging technologies cost and the increase in computing power [37] that contributed to make them more affordable and accurate. These approaches take advantage of the clear contrast between the lesion emerging on the leaf surface and the healthy leaf background. These methods, through the application of appropriate threshold, isolate lesions from coloured images (in CMYK, RGB, CIELAB, or HSV format) of the infected leaf to count their number and size in pixel. Some systems using RGB images are already available to evaluate leaf rust disease in other rust pathosystems, such oat leaf rust (*Puccinia coronata* f. sp. *avenae* Fraser & Led.) in oat (*Avena sativa* L.) [38]. More complex methods using multi- and hyperspectral sensors that collect information outside the visible light spectrum have also been developed to quantify disease severity in various pathosystem including soybean rust, wheat leaf rust, and wheat steam rust [39–41]. In particular, it has been applied to quantify leaf rust (*Puccinia triticina* Eriks.) diseases under controlled conditions in wheat (*Triticum aestivum* L.) through vegetation indices [40] and their application in the field have already been explored using unmanned aerial vehicles (UAV) [42]. However, there are currently no high-throughput image-based method that can be used to estimate rust disease evolution during the complete cycle and to estimate disease progression parameters, particularly in pea.

The growing interest in image-based disease phenotyping has driven the development of various image analysis platforms. Particularly promising are the platforms based on free and open-source environments that align with the principles of open science, with Python language being a notable example. Python’s versatility and ease of use have made it a popular choice for various scientific disciplines, including plant disease phenotyping trough packages such as PlantCV [43, 44]. In parallel, the R language, known for its extensive use in statistical computing and graphics, is also gaining traction in the field of plant phenotyping. Researchers are increasingly recognizing the capabilities of R for handling and analysing complex datasets, making it a valuable tool for studying plant diseases [45–47].

This study aimed to develop an image processing workflow using R software that achieves several goals, including producing reliable and repeatable measurements of rust-infected pea leaf area, counting the number of pustules, and measuring them on the leaf surface, combining leaf information over time to track disease progression, automating the process to analyse thousands of images, and allowing for data tracking from image acquisition to output.

## Results

### Pea rust monitoring

**Fig. 1:**
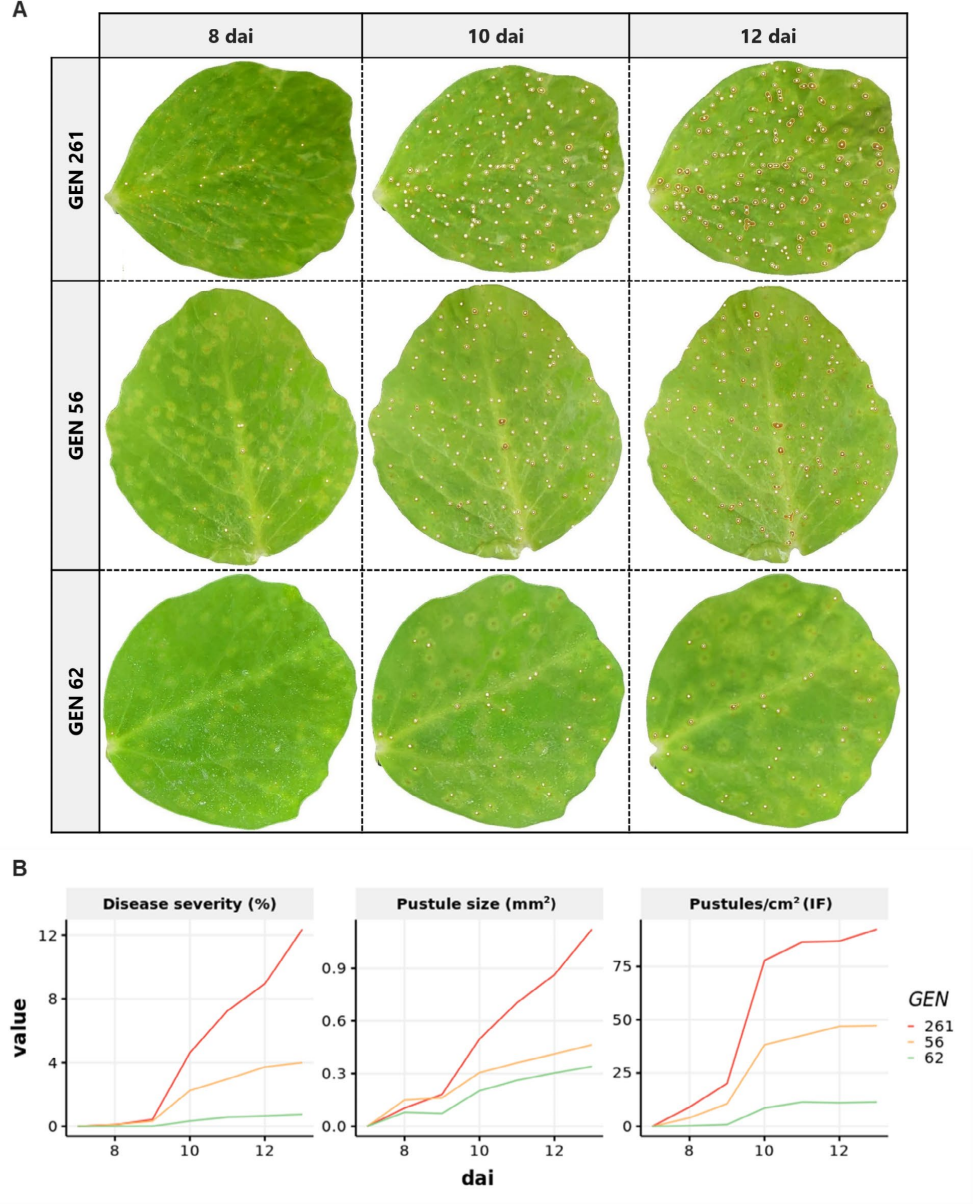
Pea rust evolution assessed with the RGB-based method. **(A)** The images show the evolution of rust pustule development at three different days after inoculation (8 dai, 10 dai, and 12 dai) on a representative leaflet of three differential genotypes (GEN62, GEN56, and GEN261) covering the wide range of susceptibility detected in the collection. White spots on the images indicate rust pustules detected by the image-based analysis methods. **(B)** Line plots showing the progression of disease severity (DS), pustule size (PS) and infection frequency (IF) over time estimated from these genotypes with the RGB-based method

The developed R script enables the tracking of rust progression through image analysis, as shown in Fig. 1A. The method allows the accurate detection of the pustules and the storage of the results in a readily usable data frame for further calculation. The evaluation of 33 diverse pea genotypes randomly selected revealed their variability in response to rust infection caused by *U. pisi*. A moderate variation was detected in disease severity (DS) which ranged from 1 to 14%. The average pustule size (PS) also exhibited variability between 0.3 and 1.0 mm², reflecting the presence of some resistance mechanism reducing the rust pustules size in some genotypes. As expected, a more pronounced variability was detected for the infection frequency (IF) that ranged from 10 to 82 pustules per cm² at 13 days after inoculation (dai) (Fig. 1B). Monitoring the evolution of these disease parameters over time showed a steady increase of DS and PS throughout the experiment (Fig. 1B), although the increment rate varies according to the genotype. IF also increment over time although in this case the increment follows an exponential evolution with a slow increase from 8 to 9 dai followed by a rapid increase from 9 to 11 dai and thereafter a saturation plateau (Fig. 1B). As for DS and PS, the increment rate of the different IF phase varied according to the genotypes. Integration of these daily disease estimates from 8 to 13 dai allowed the calculation of AUDPC, LP_50_, and MDPr progression parameters which capture most of the complexity of rust resistance and facilitate the selection and discrimination of genotypes (Additional file 1). As expected, the susceptible genotypes GEN261 exhibited the highest AUDPC and MDPr values and one of the shortest latency periods (LP_50_), while the susceptible GEN62 displayed the lowest AUDPC and MDPr values and the longest LP_50_, as expected (Additional file 1). These progression parameters, combined with daily point resistance mechanisms (IF, DS, and PS), enable a more precise estimation of the resistance or susceptibility levels of the pea genotypes to the pathogen. Similar results were obtained with the visual counting. Accordingly, these results showcase the potential of the image-based method to accurately assess rust disease progression in pea leaves and its capability to discriminate between genotypes based on their disease severity and pustule size variations.

### Processing Time optimization and RGB segmentation selection

**Fig. 2:**
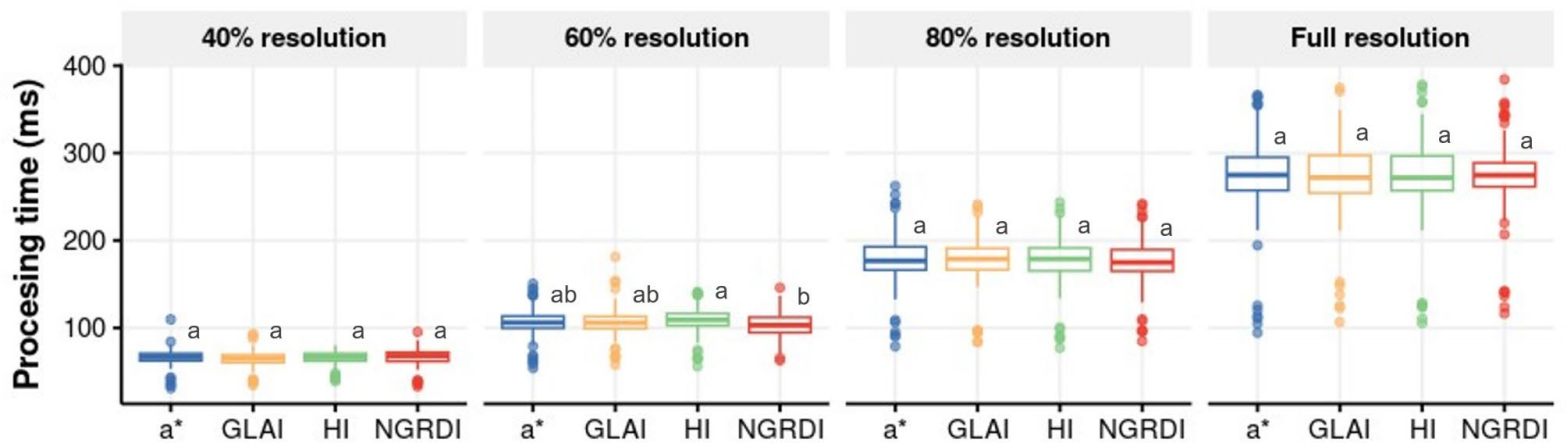
Boxplots showing the effect of image compression on processing time per leaflet by index applied to segment the pustules from healthy tissue. Different letters above the box indicate the statistical differences between indices for each image resolution estimated by Tukey HSD test at p = 0.05 for n = 600

To validate the method and select the optimal criterion, a set of 100 leaflets x 6 time-points images were randomly selected. The 600 pea samples affected by rust symptoms were analysed following a parallel or sequential batch processing approach to detect the fastest one. In all cases, parallel strategy was five time faster than sequential strategy on average (Additional file 3). Only small processing time differences was detected with the parallel strategy between segmentation index independently of the image resolution (Fig. 2). In most cases NGRDI (Normalized Green Red Difference Index) tend to be faster than the other segmentation index although the difference was only statistically significant with images at 60% resolution. At this resolution the analysis of the 600 leaflets with the NGRDI index took 62 s.

Clear differences in processing time were observed between compression levels. The processing time required to analyse a single leaflet image varied from an average of 65.7 ms at 40% resolution to 274 ms at full resolution (Table 1). Therefore, reducing the image resolution allowed decreasing processing time from up to 76% at 40% resolution (Table 1). The image compression also allowed reducing storage space required in the ROM memory. The average input image size in megabytes (Mb) varied from 3.5 to 1.0 between full resolution to 40% resolution, respectively, resulting in a store saving of up to 71% at maximum compression in comparison to full resolution (Table 1).

**Table 1:** Effect of image compression over processing time and image size

Resolution	Time by leaflet (ms)	Input Image size (Mb)	Speed increase vs. full resolution (%)	ROM saving vs. full resolution (%)
full (3024 px)	274	3.5	-	-
80% (2419 px)	178	3.1	35	11
60% (1814 px)	106	2.0	61	43
40% (1210 px)	65.7	1.0	76	71

**Fig. 3:**
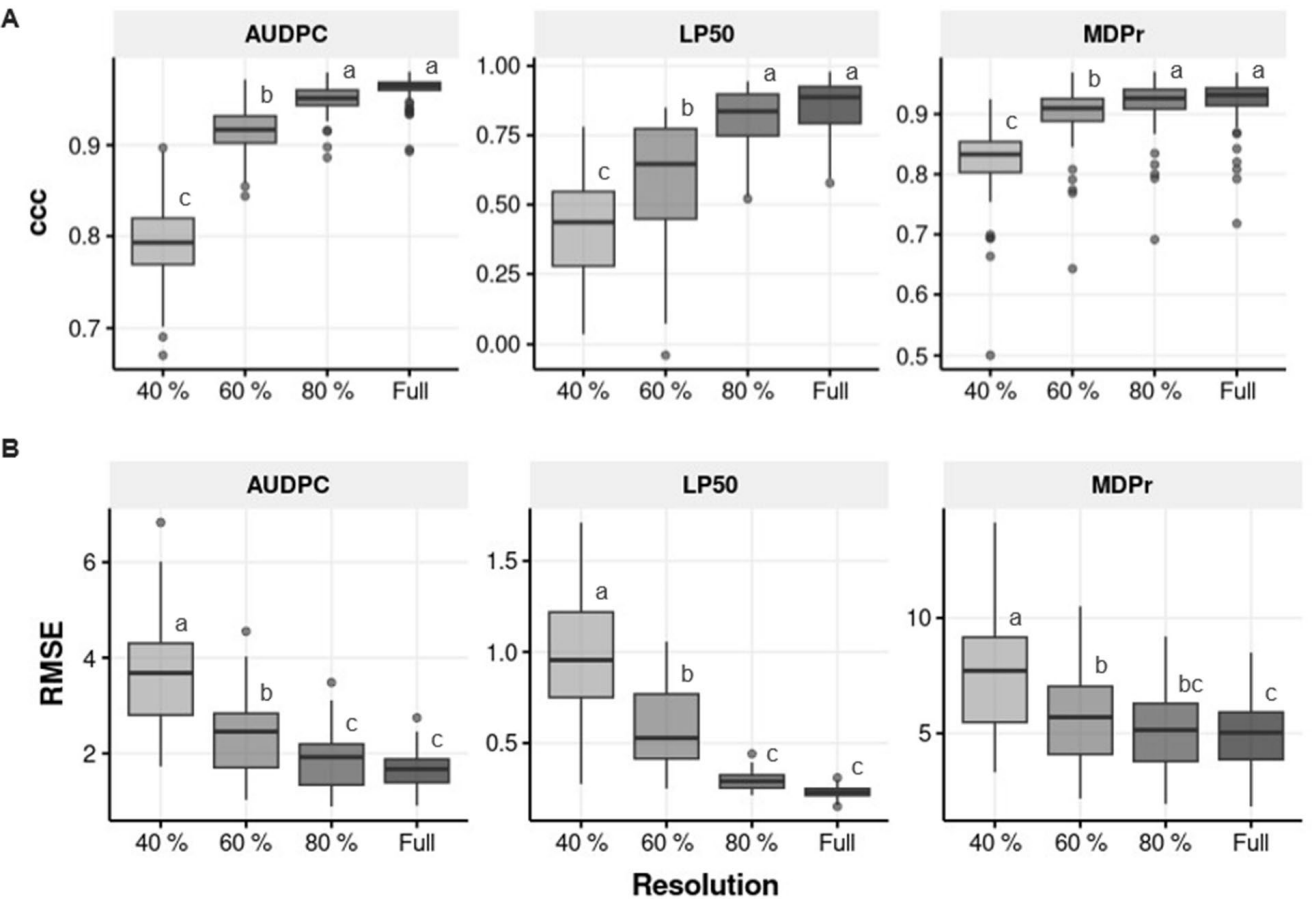
Boxplots showing the effect of image compression in the precision of comparison, by accuracy **(A)** and RMSE **(B)**, between visual calculation and image-based calculation on LP_50_, MDPr and AUDPC. Different letters above the boxes indicate statistically significant differences at *p = 0.05* according to the Tukey HSD test for n = 600

To select the most appropriate compression level without compromising accuracy of rust pustule estimation, concordance correlation coefficient (ccc) using a resampling approach were evaluated between visual pustule counting and image-based analysis. As expected, the averaged indices ccc and RMSE varied largely depending on the compression level. As expected, accuracy for all traits was proportional to the image compression level (Fig. 3A) while RMSE was inversely proportional to image compression level (Fig. 3B). The highest accuracies and lowest RMSE were always obtained at full resolution. However, the accuracies and RMSE obtained for all traits at 80% resolution were not statistically different to the full resolution (Fig. 3). At these resolutions, the accuracy of AUDPC and MDPr estimates ranged from 0.952 to 0.962 for AUDPC and from 0.918 to 0.922 for MDPr. LP_50_ was more difficult to estimates with accuracies varying from 0.811 to 0.852. Increasing the compression level reduced accuracy and increased RMSE although accuracies of AUDPC and MDPr estimations at 60% resolution was still higher than 0.9 (ρc = 0.918 and ρc = 0.901 for AUDPC and MDPr, respectively) (Fig. 3).

**Fig. 4:**
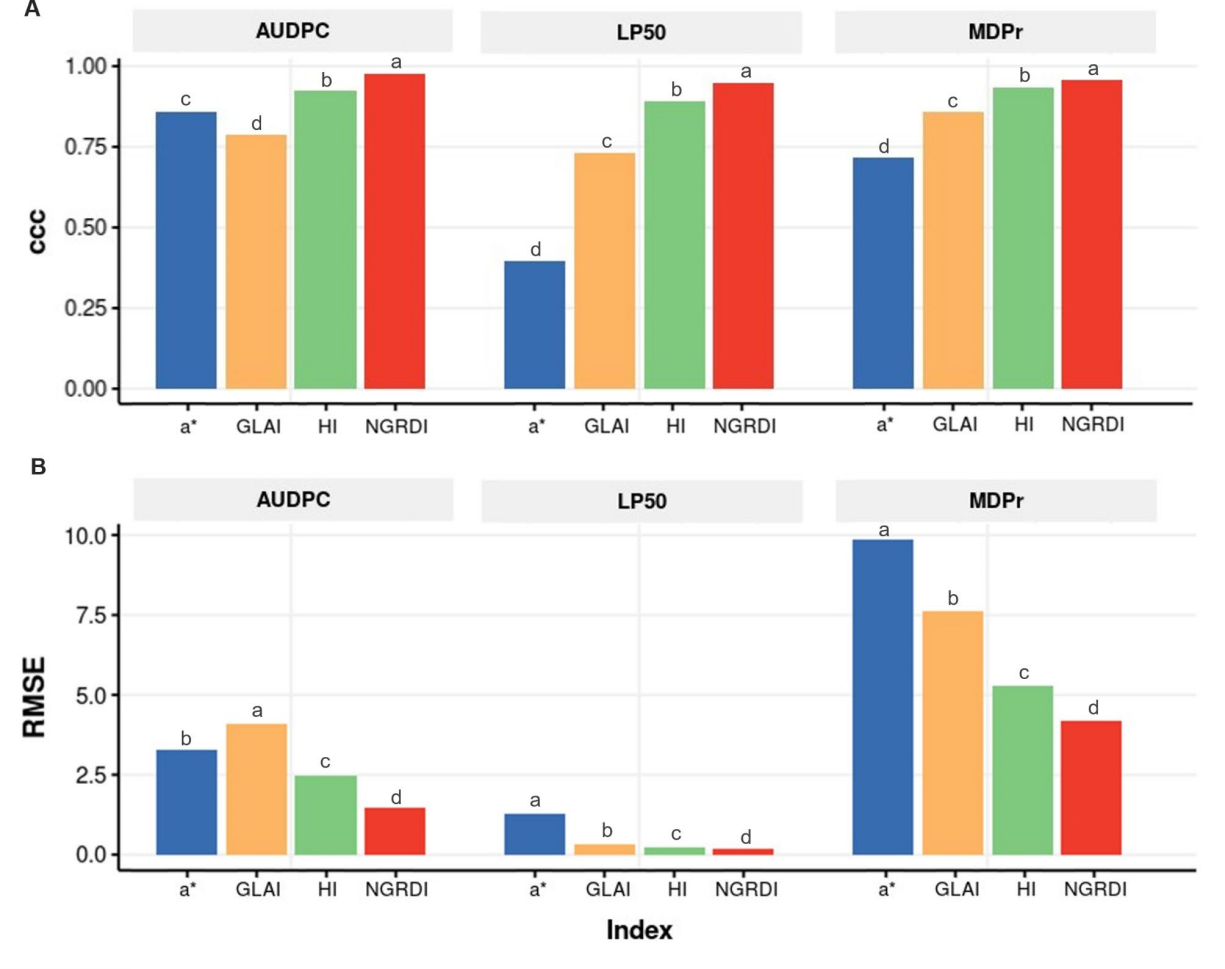
Bar plots showing the effect of the different indices on accuracy **(A)** and RMSE **(B)** when visual method and image-based method are compared by each parameter studied from images at 60% of the full resolution. Different letters above the boxes indicate statistically significant differences at *p = 0.05* according to the Tukey HSD test for n = 600

**Fig. 5:**
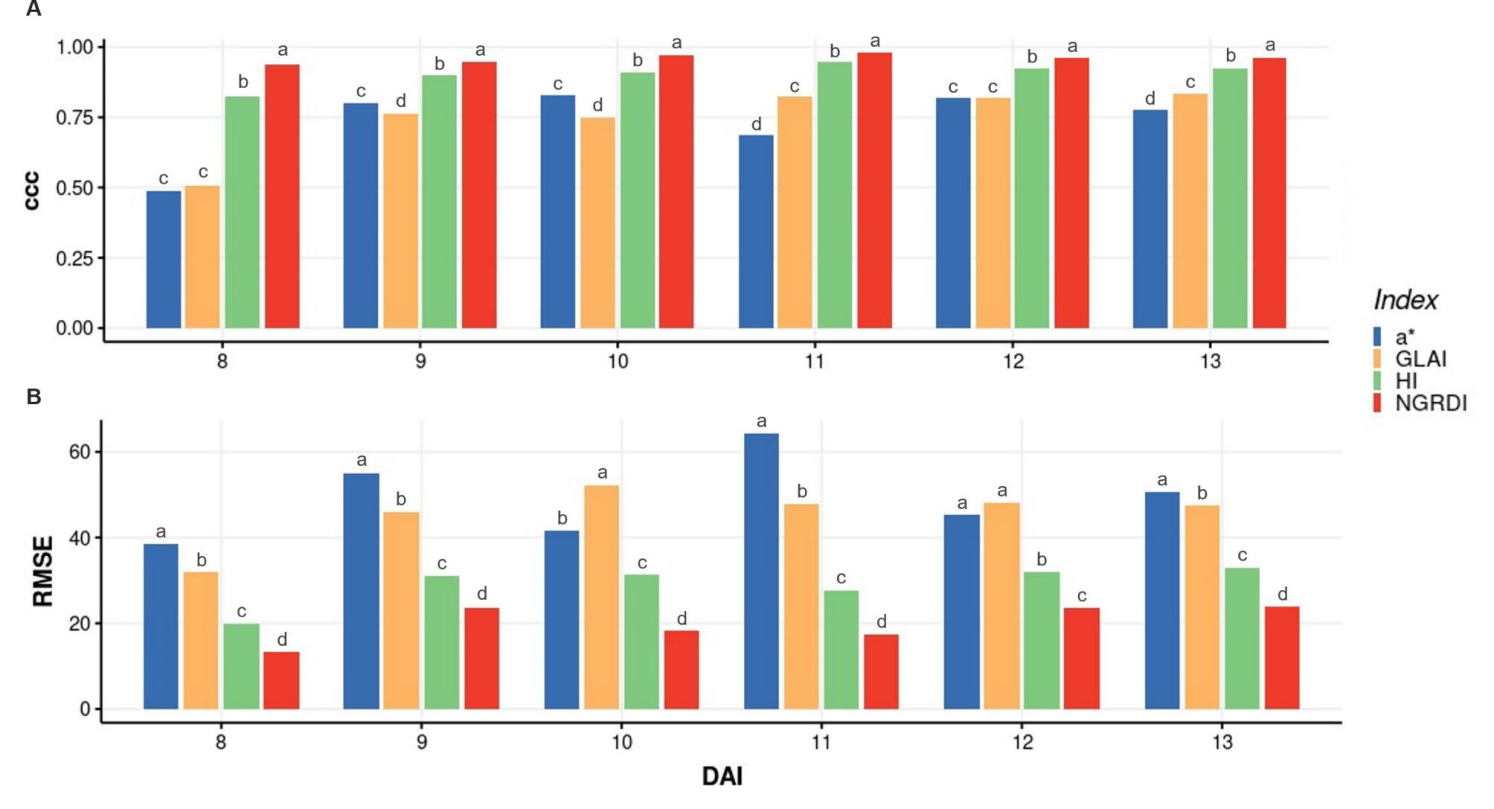
Bar plots showing the effect of the different indices on accuracy **(A)** and RMSE **(B)** when visual method and image-based method are compared for each parameter studied at 60% resolution. Different letters above the boxes indicate statistically significant differences at *p = 0.05* according to the Tukey HSD test for n = 600

Significant differences in accuracy and RMSE were also detected between segmentation index for all estimated disease parameters (Figs. 4 and 5). In all cases, the NGRDI index was the best index accumulating significantly less error and providing a significantly higher accuracy while a* chrominance from LAB colour space and GLAI (Green Leaf Area Index) were the worst. The average accuracies of NGRDI were 0.975, 0.945, and 0.957 for AUDPC, LP_50_, and MDPr, respectively. The average accuracy of HI (Primary Colours Hue Index) was also higher than 0.9 in all cases, suggesting that this index also provided suitable rust estimation, may be useful to analyse leaves from other species.

Variations were also detected in the estimation capacity of each model over time. Indeed, accuracy and RMSE obtained from the estimations obtained from the different indices were more variable at 8 and 9 dai then at later stages (Fig. 5). In general, accuracy increase while time advances and RMSE decrease. NGRDI was the only index which gave accuracies higher than 0.9 for all time points, reaching an accuracy of 0.98 at 11 dai. Although accuracy of HI was slightly lower, the estimation capacity of HI was still acceptable (Fig. 5).

## Discussion

In recent years, advancements in image analysis software and computing power have enabled the use of high-throughput methods for plant disease phenotyping. These methods are nowadays used to analyse plant diseases at different architecture levels, including stems, leaves, and roots [37]. Remote sensing techniques are playing a major role in modern breeding programs, providing accurate and high-resolution methods for identifying and quantifying novel natural variations within crops [48–50]. This present study describes a new method for the automatic assessment of daily rust disease parameters from RGB images and their integration into rust disease progression parameters fastening both disease ratings and phenotype data analysis. The method, developed on the R programming environment, counts, measures, and reports the damage caused by rust on pea leaflets. Moreover, when images are provided in a temporal sequence, the method can accurately integrate the damage into the most common disease progression parameters and report them by genotype in a ready-to-use data frame. Overall, the image-based method proposed here to analyse rust disease progression in pea provides breeders with a powerful tool to improve the efficiency and effectiveness of their breeding programs. It enables the rapid and accurate screening of large germplasm collections against rust, which will facilitate the future development of pea cultivars with high level of rust resistance. Although not tested, the method proposed should be easily applied to evaluate rust in other plant species.

## Automatization of pea rust progress monitoring

Traditional image-based methods for evaluating plant aerial diseases have been destructive and do not allow comprehensive disease tracking. The proposed method enables the periodic evaluation of several disease parameters throughout the first cycle of rust disease on the same sample and to integrated them into disease progression parameters (AUPDC, MDPr and LP_50_) providing a comprehensive analysis of the pea genotype response to rust. This approach is an adaptation of previously designed detached leaf assay used to assess other foliar diseases such as powdery mildew in legumes [51] and cereals [52] which enable the preservation of viable leaflet simples throughout the first cycle of rust disease and ensure standardize condition for image acquisition. One of the key advantages of the proposed method is the improved efficiency in data collection. The image analysis workflow (Additional file 2) allows for disease monitoring and captures maximum information regarding disease progression in an automatic process, which, as far as we know, could not be achieved by the previously developed methods [38, 50, 53, 54]. Here, estimation of the daily resistance components (IF, DS, and PS) for each leaflet allows the calculation of disease progression parameters such as AUDPC, LP50, and MDPr for each genotype. Application of this method was suitable to discriminate between genotypes and identify pea genotypes with high partial resistance such as GEN62 (Fig. 1) providing seminal works for the implementation of this method to evaluate large pea collection. The fast, accurate and comprehensive information gathered by this method is crucial for future breeding efforts of pea with higher resistance to rust [6].

Very few image-based analysis methods tackle temporal analysis of fungal infection in plants [55, 56]. Beside some studies in different *Arabidopsis thaliana* (L.) pathosystems [55], Only one study targeted rust and compared rust disease progression parameters estimated by image analysis in R or visual rating [57]. This study that counted rust pustules on ryegrass leaves with the “*EBImage*” R package allowed to estimate AUDPC with an accuracy of 0.77 which is lower than the accuracy we obtain in pea with the present method (ρ_c_ = 0.975). In addition, by contrast with all previous method, calculation of disease progression parameters is integrated in the R script, resulting in an automated process that incorporates all quantitative assessments obtained through RGB image analysis that will help researchers to better understand disease progression and resistance mechanisms in aerial diseases.

## Processing time optimization

Image-based disease assessments face the challenge of balancing storage capacity, processing time, and accuracy. The present R-based approach is based on the “*pliman*” package functions. This package, recently launched by Olivoto et al., is specifically designed for plant disease image phenotyping [47]. It is a promising tool faster than other software such as the widely used license-based APS Assess 2.0 software or the LeafDoctor free-app, while still maintaining high levels of accuracy [58]. The processing speed of the R package “*pliman*” has been considerably increased compared to the first stable version available on CRAN (v 1.0.0). For example, the processing time required to analyse one image of ~ 3 mega-pixels (1367 × 2160) with only one leaflet was previously reported as ~ 1 and 3 s, for a parallel and sequential strategy, respectively [58]. Considering the average time to process one Petri dish (~ 900 ms) with 9 leaves using an image of ~ 3.3 mega-pixels (1814 × 1814), we have shown that the processing time per leaflet is almost nine time faster. The greater speed observed here is attributed to recent improvements of the packages that now use C + + language for the most critical functions [59] which offers faster computation speeds compared to other languages such as JavaScript or Python [60]. The potential of “*pliman*” to quantify disease severities was initially explored on infected *Populus* spp. leaves, and it was found to be faster and more efficient than manual analyses with ImageJ software to estimate necrotic area percentages [61]. However no previous studies used “pliman” to assess rust disease.

The present method can analyse 100 pea samples in 27.4 segs at full resolution, or in 10.6 segs at 60% resolution, provide estimates with accuracies higher than 0.91 at all time-points. This is a significant improvement compared to the previously developed RUST software developed on Image-J that took 20 to 80 min to estimate IF on 100 oat samples in automatic and semi-automatic mode, respectively [38] [62]. Additional methods using free or licensed image-based analysis software are able to predict rust IF with good accuracy. Although not all studies reported processing time. The present method appears, as far as we know, 100 to 200 times faster than previously existing method to quantifying rust IF. In addition, these previous methods did not allow estimation of disease progression parameters such as AUDPC while they are automatically estimated by the present method within the processing time. In others pathosystems, incorporation of additional colour space transformations or implementation of machine learning tool was shown to improve lesion segmentation and accuracy however each additional step increased processing time. For example, McDonald et al. proposed an automated method for measuring soybean [*Glycine max* (L.) Merr] frogeye leaf spot that involves converting RGB images to HSB (hue, saturation, brightness) and then to LAB (lightness, a* chrominance, b* chrominance) to remove the background and isolate the lesion [34]. While the method was highly accurate, reaching accuracy of 0.99 it took 16.7 min to analyse 100 leaf samples. Although, this method was slightly more accurate, it was around 100 times slower than the method proposed here. Implementation of machine learning to segment and quantify cassava (*Manihot esculenta* Crantz) bacterial blight disease severity also improved accuracy but takes 250 min to analyse 100 cassava leaves due to higher computer requirements [63]. The method proposed here is simpler and more cost-effective allowing the comprehensive fast analysis of pea rust disease without compromising accuracy. It is based on RGB spectral indices segmentations discussed by Alves et al. [54]. These authors also coincide in the use of NGRDI and HI as the optimal one for foliar diseases segmentations when compared to others [54].

The high accuracy provided for all disease parameters compared with the present method coupled with it unprecedent speed which should be even more reduced by reducing image resolution to 60% if needed allow it is implementation to evaluate large collections. It could be the method of choice for the evaluation of NAM population, typically comprising several thousand genotypes [21, 23] that cannot be evaluated by current rust evaluation methods.

### Rust resistance mechanisms estimations through RGB images

The proposed RGB image-based method in controlled conditions showed high accuracies (ρc) exceeding 0.9, and in most stages of the disease cycle. This method requires neither a large budget nor specific training, making it a cost-effective and feasible option for phenotyping rust in pea and other crops. In contrast to other complex techniques like multi- or hyperspectral imaging, which have also proven useful in rust phenotyping in different rust pathosystems [64, 65], our approach stands out as a more accessible and user-friendly alternative. The acquisition of these sophisticated phenotyping platforms can be prohibitively expensive and demands specialized training, limiting their widespread application [66].

Traditionally, DS is the measure used to assess the extent of damage caused by plant diseases, especially those affecting the aerial parts [54]. The colour thresholding method used in this study is considered the most reliable method to accurately determine DS in phytopathometry [67]. To automate DS assessment, the capacity to accurately predict DS of several free open-source or licensed software have been explored [36, 68–70]. In the context of the R environment, Mattos et al. [57] developed models that indirectly determined the percentage of injured area from images of septoria leaf spot in tomatoes (*Solanum lycopersicum* L.), achieving accuracies of 0.925 and 0.98 for the percentage of necrotic area and the necrotic plus chlorotic area, respectively. These accuracies are similar to the accuracies obtained with the present method, but the method proposed by Mattos et al. required to manually delimit the injured area using an image software (GIMP) which is not require for our method. Previous study on crown and stem rust in perennial ryegrass (*Lolium perenne* L.), using the “EBImage” R package predicted crown rust with similar accuracy (0.93) but only allow evaluation of from single-leaf samples at a single time point and it was around 10 time slower [56].

Despite being widely used, visual DS estimations can be imprecise and biased for diseases with small and numerous lesions like rust [25]. Therefore, researchers usually also analyse IF and/or PS that are less prone to user bias to quantify more precisely partial resistance in pea [71]. Some previous studies reported the estimation of some of these disease components through RGB image analysis with variable efficiency [38, 62, 72, 73].

For instance, the widely used license based Assess 2.0 software seems efficient to estimate rust PS in wheat [72] although its accuracy to estimate IF was more limited as shown by a study on maize (*Zea mays* L.) leaves (R^2^ = 0.49) [73]. By contrast, highly accurate estimation of rust IF in oat was obtained with the license-based Image Pro or the free ImageJ softwares that reported accuracies of 0.97 in pustules counting but with image resolution doubling the image resolution required by the present method [38][62]. Heineck et al. also estimate IF in their methodology of crown and stem rust in perennial ryegrass images, although the reported accuracies were lower reaching 0.77 and 0.84 respectively [56].

Moreover, our image-based approach provides a more detailed and precise characterization of rust disease resistance mechanisms and its progression. Traditional methods may suffer from subjectivity and limitations in capturing subtle variations in rust disease development. In contrast, our method captures the high complexity of rust disease by analysing daily symptoms and integrating them into disease progression parameters, allowing for a more comprehensive understanding of the plant-pathogen interaction.

## Conclusions

Accurate and detailed information on the phenotype of rust disease in pea crop is crucial to develop new cultivars with improved genetic resistance. The proposed method for image-based rust phenotyping uses RGB spectral indices segmentation and pixel value thresholding to separate important features from the image, such as the leaf and pustule lesions if present. This enables the measurement of disease severity by calculating the percentage of the leaflet area affected and counting the number of pustules on a leaflet. With minimal computational requirements, the program can analyse hundreds of images in seconds and has accuracy comparable to visual methods. The proposed method is significantly faster than previously developed image-based workflows for plant disease phenotyping without compromising accuracy. In addition, this is the first methods that allow to capture most of the complexity of rust disease in pea by assessing daily DS, IF and PS and integrating them into three disease progression parameters through an automated process. Being developed as an R script, the proposed method can also easily adjust to evaluate rust in other pathosystems where these detailed measurements are necessary to comprehend partial disease resistance. In addition, the application of image processing alleviates the raters bias that can be introduce in traditional methods, making it a convenient and precise approach to gather data on rust disease symptoms. As a results, application of the proposed method can have implications for both basic research and plant breeding, paving the way for more effective disease management strategies and the development of pea varieties with higher resistance in the future.

## Methods

### Plant materials, Pathogen isolate and Inoculation

The plant material used in the image analysis to set up and validate our method was a randomly selected subset of 33 accessions from a pea core collection of 320 genotypes which previously reported to show a wide variability of responses to rust caused by *Uromyces pisi* [74]. Disease assays were performed at seedling stage under controlled conditions. The experiment followed a randomized complete block design with three biological replicates being planted at a time, using pea cv. Messire as a high-susceptible rust control, meaning a total of 100 experimental units. Seeds of each accession were surface-sterilized, scarified and vernalized to ensure optimal germination. Three germinated seeds per accession were sown in a sand:peat mixture (1:1, w/w) in a 15 cm^2^ plastic pot. At 7 days post germination, plants were thinned to one plant per pot to maximize light distribution. The growth chamber was maintained at 20 ºC with a photoperiod of 14 h of light and 10 h of darkness and 148 µmol m^− 2^ s^− 1^ of irradiance at plant canopy level. Once the third leaf of each plant was fully expanded, plants were inoculated with freshly collected urediospores of the highly virulent isolate UpKeS-05 of *U*. *pisi* [7] previously multiplied on cv. Messire seedlings. Inoculation was performed by dusting the plants with 1 mg urediospores per pot, mixed in pure talc (1:10, v:v) and the infected plants were incubated for 24 h at 20 ºC in complete darkness and 100% relative humidity as previously described [75]. Then, plants were transferred back to the growth chamber. Pustules associated with rust symptoms started to emerge on pea plants eight days after inoculation (dai).

### Image Acquisition

**Fig. 6:**
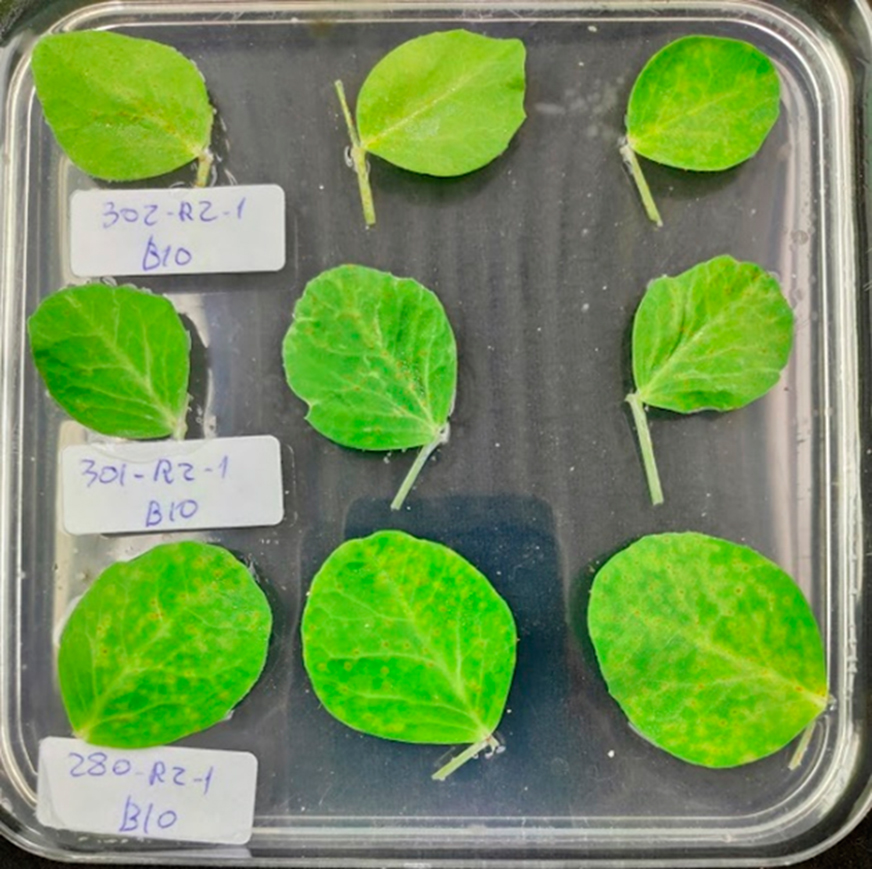
Example of a stored RGB image. The image represents a typical Petri dish containing nine inoculated pea leaflets and their labels. Each row contains the same genotype, and the columns are their biological replicates. The example shows the genotypes 302, 301, and 280 at 8 dai

In order to cover the first rust disease cycle that goes from 8 to 13 dai, a leaflet from the third leaf of each plant (n = 100) was cut at 7 dai and transferred to square Petri dishes filled with water:agar (0.5%) media and 0.005% Benzimidazole as fungicide with nine leaflets per Petri dish (Fig. 6). Petri dishes were maintained in the growth chamber until the end of the experiment. RGB images of the whole Petri dishes were then acquired daily from 8 to 13 dai, with a smartphone brand Xiaomi, carrying a Sony IMX363 Exmor RS Sensor with a focal ratio ƒ/ 1.9 with a 12-megapixel resolution. To ensure homogeneity of the RBG images, the smartphone was set on a tripod 0.35 m above the Petri dish and images were acquired on a plain black background under fluorescent light tubes set at 35º angles on both side of the plate. White balance, shutter speed, aperture and ISO speed of the camera was adjusted according to default parameters without flash. Each Petri dish was opened before image acquisition to avoid light reflection and closed thereafter to prevent contaminations (Fig. 6). Daily RGB images were saved in .*jpg* format with an original resolution of 3024 × 3024 pixels.

### Disease assessments

Infection frequency (IF) was estimated by counting the number of rust pustules emerging daily on each leaflet from 8 to 13 dai, visually or through the image analysis procedure. The resulting daily counting were then integrated in three parameters representing disease progression:

- AUDPC. The Area Under Disease Progress Curve [76] following the formula:

$$AUDPC= \sum _{i=1}^{n-1}\frac{{y}_{i}+{y}_{i+1}}{2}\times \left({t}_{i+1}-{t}_{i}\right)$$

where y_i_ is the IF at the ith observation, t_i_ is days at the ith observation, and n is the total number of observations.

- MDPr. The Monocyclic Disease Progress rate, as described by Arneson (2001) [33], is a proportionality constant that represents the rate of disease progress per unit of inoculum.
- LP_50_. The Latency Period is the elapsed time between inoculation day and the day when 50% of pustules are formed.

### Image segmentation

Image-based quantification of rust damage requires a two-stage segmentation of the original images to separate leaves from the background and to distinguish rust damage from healthy tissue. This segmentation was performed for each image subset allowing the estimation of DS, IF and PS.

The colour differences between foreground and background in our images are represented by different values from the red (R), green (G), and blue (B) channels in the RGB colour space, allowing the object segmentation in the images. Accordingly, the first segmentation was performed by applying a HUE index prevailing the green region to isolate the leaflets from the background with the formula:

$$HUE= \frac{{atan}\left(2\left(B-G-R\right)\right)}{30.5(G-B)}$$

The threshold used to separate the background from the leaflets were based on the Otsu method [77]. To isolate rust pustules from the healthy tissue, a second segmentation step was performed. Four indices commonly used in remote sensing and phytopathometry were tested for their capacity to detect rust pustules [78–82], three operating in the RGB channels and one in the CIELAB colour space stack. The selected indices were the Normalized Green Red Difference Index ($NGRDI= \frac{G-R}{G+R}$ ) [83], Primary Colours Hue Index ($HI= \frac{2(R-G-B)}{G-B}$), Green Leaf Area Index ($GLAI= \frac{25(G-R)}{\left(G+R-B\right)+1.25}$), and the a*- chrominance channel from CIELAB band (${a}^{*}= 0.55\frac{(R-\left(0.2126 R+0.7152 G+0.0722 B\right))}{1-0.2126}$). Each index applies a different transformation of RGB values, therefore, each one requires a different threshold. The thresholds used were set as 0, 1, 1, and 0.50 for NGRDI, HI, GLAI, and a*- respectively. To maximize the accuracy of image-based pustules counting, the Watershed algorithm was also implemented, permitting to segment pustules connected by a few pixels that could be considered as two distinct lesions [84].

### Image Compression and Processing Time

Image compression can improve the processing time while saving store capacity in RAM and ROM memories. To get the optimum processing time without compromising precision and accuracy, four different compression levels ranged from full resolution (3024 × 3024 pixels) to 40% of the full resolution (1210 × 1210 pixels) were tested. Images compression were performed by applying the image_resize() function from “*pliman*” R package [47]. The processing time required to analyse all images at each compression level was calculated using mark() function from “*bench*” R package [85].

### Method validation

To validate and select the best segmentation index and optimum image compression, the Lin’s concordance correlation coefficient (ccc, ρ_c_) [86] was computed between visual counting and software-estimation for each dai (from 8 to 13) and disease progression parameter (AUDPC, MDPr and LP_50_). The Lin’s ccc not only evaluates how well the software-predicted values align with the visual counting values but also considers their systematic differences and scale variations. It provides a comprehensive assessment of the agreement by measuring both the correlation and the bias between the predicted and real values. Therefore, the ccc has been widely recommended and utilized in studies that involve comparing estimated severity values with actual severity values in phytopathometry [25][87][88]. This parameter was calculated using a resampling approach between predicted and visual values for each parameter using the R package “*yardstick*” [89]. In addition, the root-mean-square error (RMSE) were also calculated for an additional accuracy estimation between visual and software-based calculations. ρ_c_ values range from 0 to 1 while RMSE values are in the same units as the original data.

### Description of rust evaluation method

The script controlling the image analysis method was developed in R software version 4.2.2 [90] under RStudio version 2022.07.2.576, using the R packages “*pliman*” [47], “*EBImage*” [91], and “*Tidyverse*” [92]. The approach to analyse the images using batch processing were also implemented with the R package “*foreach*”, which facilitates to compute the analyses in a parallel process. This parallel strategy allows to split the jobs across multiple cores in the CPU, regarding an extra processing time saving. All the analyses were performed on a PC equipped with an AMD Ryzen 9 CPU (16 cores) with 3.4 GHz frequency, a NVIDIA GTX 1660 Ti GPU, and 32 GB of RAM memory.

The method was developed using the set of 600 individual leaflets with different levels of rust disease symptoms. The symptoms ranged from no disease to leaflets heavily covered with pustules. The image processing pipeline (Additional file 2), developed as a R script [93], consists of a function which imports and analyses the images following a batch processing strategy. First, the input plate image is loaded following a name pattern inside the file path, then it is resized, before the nine leaflets within plate image are split according to the first index segmentation (HUE index). Then the split samples are analysed individually through a *for loop* using the measure_disease() function that estimates the total leaflet area, the number of pustules, the leaflet area covered by pustules and the mean pustule size, and saves these values for each sample in the output data frame. Furthermore, the developed function integrates an additional argument to analyse the input images in parallel (“parallel” argument set to “TRUE”) or sequentially (if parallel argument set to “FALSE”).

Therefore, the method can rapidly and accurately count the number of pustules (IF), report the percentage of leaf area covered by pustules (DS) and the average pustules size (PS) for each genotype daily from 8 to 13 dai. The daily values obtained for the same leaflet/genotype are then stored and combined into the AUDPC, MDPr and LP_50_ parameters, also stored in the output data frame.

### Electronic supplementary material

Below is the link to the electronic supplementary material.

Additional file 1. Histograms showing disease parameters distributions. Red, yellow, and green arrows indicate the values for GEN261, GEN56 and GEN62, respectively.

Additional file 2. Image processing pipeline. **(A)** shows the image modifications from the original image to the individual leaflet output and **(B)** represents the function flowchart summarized in the script. Every coloured region represents the four main steps. In green, the image loading; in yellow, the leaflet segmentation; in blue, the lesion segmentation and, in grey, the storing of the collected data and reporting.

Additional Table 3. Table 1. This table shows the processing time for 600 leaflets of the CPU in hh:mm:ss format by index, processing strategy and resolution applied.

## Data Availability

The datasets generated during and/or analysed during the current study are available in the Zenodo repository, 10.5281/zenodo.7991462 [93].

## List of abbreviations

a*: values relative to the green–magenta opponent colours in the CIELAB colour space
AUDPC: Area under the disease progress curve
b*: values which represent the blue–yellow opponent colours in the CIELAB colour space
ccc: the Lin’s concordance correlation coefficient
CIELAB: Three-dimensional colour space defined by the International Commission on Illumination
CS: Colony size
dai: days after inoculation
DS: Disease severity
GLAI: Green Leaf Area Index
HI: Primary Colours Hue Index
IF: Infection frequency
IT: Infection type
L*: values referred as perceptual lightness in the CIELAB colour space
LP_50_: Latency period
MAS: Marker-assisted selection
MDPr: Monocyclic disease progress rate
NGRDI: Normalized Green Red Difference Index
PS: Pustule size
RGB: Red – Green - Blue
RMSE: Root-mean-square error
SAD: Standard area diagram
UAV: Unmanned aerial vehicle
ρ_c_: Lin’s concordance correlation coefficient
