# Supplementary material for: RGB image-based method for phenotyping rust disease progress in pea leaves using R
Source: Plant Methods. 2023 Aug 21;19:86. doi: 10.1186/s13007-023-01069-z (PMC10440949; doi:10.1186/s13007-023-01069-z)
Supplement: Supplementary file 1 — Additional file 1. Histograms showing disease parameters distributions. Red, yellow, and green arrows indicate the values for GEN261, GEN56 and GEN62, respectively. [file 13007_2023_1069_MOESM1_ESM.pdf]

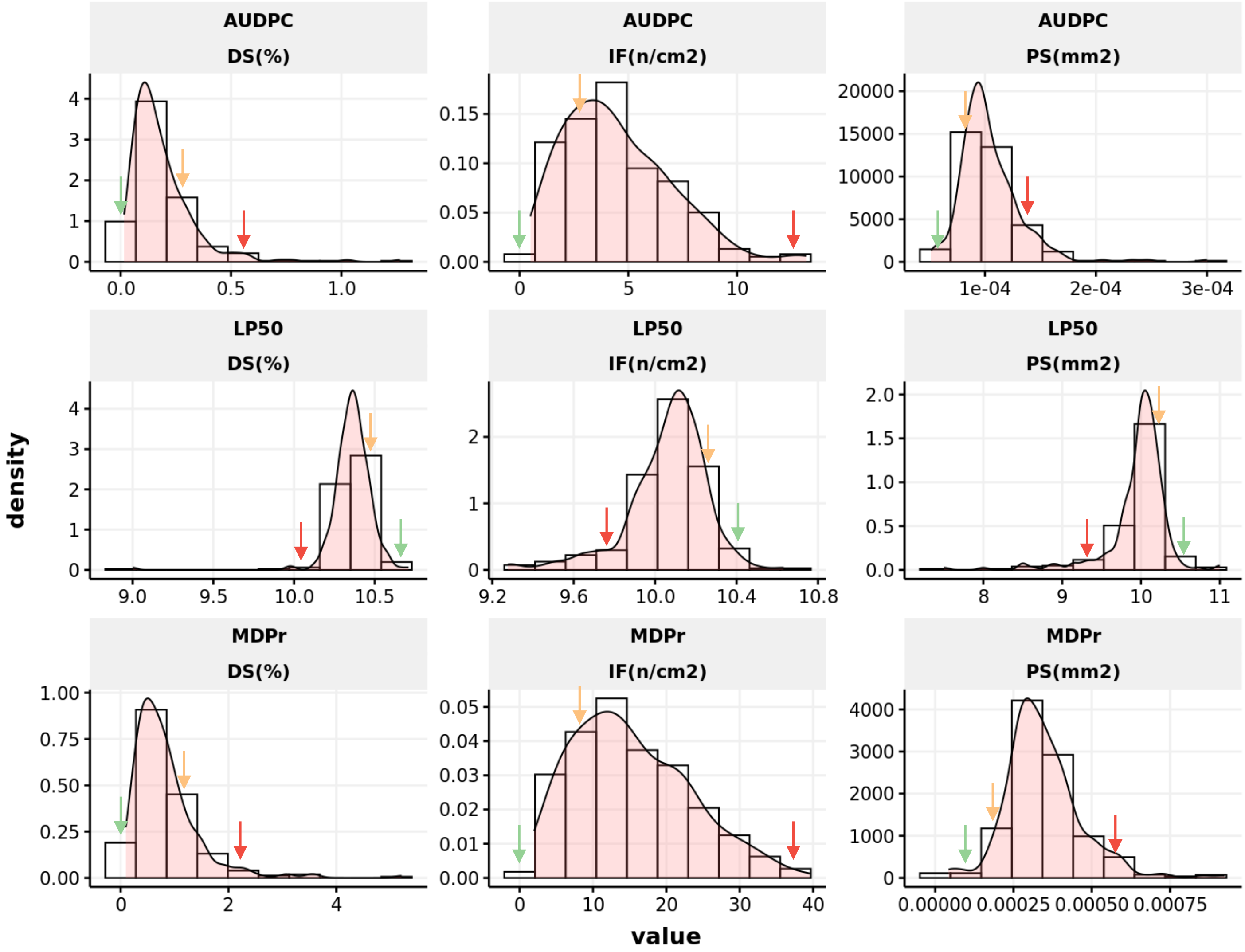

Additional file 1. *Histograms showing disease parameters distributions. Red, yellow, and green arrows indicate the values for GEN261, GEN56 and GEN62, respectively.*
