# Supplementary material for: RGB image-based method for phenotyping rust disease progress in pea leaves using R
Source: Plant Methods. 2023 Aug 21;19:86. doi: 10.1186/s13007-023-01069-z (PMC10440949; doi:10.1186/s13007-023-01069-z)
Supplement: Supplementary file 2 — Additional file 2. Image processing pipeline. (A) shows the image modifications from the original image to the individual leaflet output and (B) represents the function flowchart summarized in the script. Every coloured region represents the four main steps. In green, the image loading; in yellow, the leaflet segmentation; in blue, the lesion segmentation and, in grey, the storing of the collected data and reporting. [file 13007_2023_1069_MOESM2_ESM.pdf]

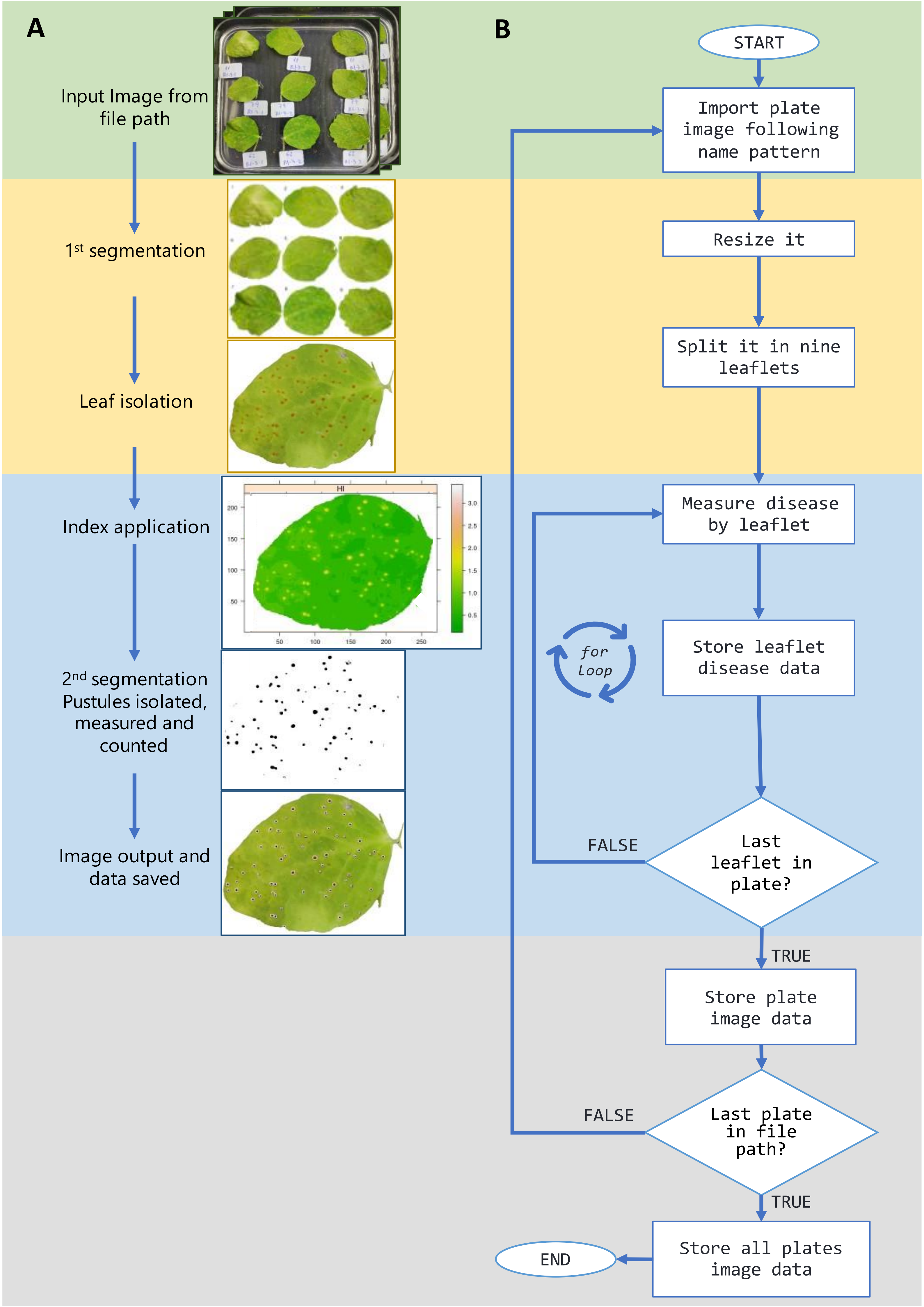

A
Input Image from file path
1st segmentation
Leaf isolation
Index application
2nd segmentation Pustules isolated, measured and counted
Image output and data saved
B
START
Import plate image following name pattern
Resize it
Split it in nine leaflets
Measure disease by leaflet
Store leaflet disease data
for Loop
Last leaflet in plate?
FALSE
TRUE
Store plate image data
Last plate in file path?
FALSE
TRUE
Store all plates image data
END

***Additional file 2.*** *Image processing pipeline. A shows the image modifications from the original image to the individual leaflet output and B represents the function flowchart summarized in the script. Every coloured region represents the four main steps. In green, the image loading; in yellow, the leaflet segmentation; in blue, the lesion segmentation and, in grey, the storing of the collected data and reporting.*
