# Supplementary material for: RGB image-based method for phenotyping rust disease progress in pea leaves using R
Source: Plant Methods. 2023 Aug 21;19:86. doi: 10.1186/s13007-023-01069-z (PMC10440949; doi:10.1186/s13007-023-01069-z)
Supplement: Supplementary file 3 — Additional Table 3. Table 1. This table shows the processing time for 600 leaflets of the CPU in hh:mm:ss format by index, processing strategy and resolution applied. [file 13007_2023_1069_MOESM3_ESM.pdf]

			Pustules index segmentation			
			NGRDI	HI	GLAI	a*
**Processing strategy**	Sequential	Full resolution	00:12:40	00:12:55	00:12:27	00:12:10
		80 % resolution	00:08:23	00:08:53	00:08:21	00:08:13
		60 % resolution	00:05:18	00:05:44	00:05:10	00:05:06
		40 % resolution	00:03:06	00:03:19	00:03:19	00:02:58
	Parallel	Full resolution	00:02:22	00:02:27	00:02:26	00:02:25
		80 % resolution	00:01:39	00:01:40	00:01:40	00:01:39
		60 % resolution	00:01:02	00:01:09	00:01:05	00:01:05
		40 % resolution	00:00:42	00:00:42	00:00:42	00:00:42

Additional table 1. *This table shows the processing time for 600 leaflets of the CPU in hh:mm:ss format by index, processing strategy and resolution applied.*
